# CRIPTO overexpression promotes mesenchymal differentiation in prostate carcinoma cells through parallel regulation of AKT and FGFR activities

**DOI:** 10.18632/oncotarget.2740

**Published:** 2015-01-22

**Authors:** Stéphane Terry, Ihsan Y. El-Sayed, Damien Destouches, Pascale Maillé, Nathalie Nicolaiew, Guillaume Ploussard, Fannie Semprez, Cynthia Pimpie, Himisha Beltran, Arturo Londono-Vallejo, Yves Allory, Alexandre de la Taille, David S. Salomon, Francis Vacherot

**Affiliations:** ^1^ Inserm, U955, Equipe 7, Créteil, France; ^2^ Université Paris-Est, UMR_S955, UPEC, F-94000, Créteil, France; ^3^ Institut Curie, Centre de Recherche, CNRS UMR 3244, Paris, F-75248, France; ^4^ Inserm, U753, Institut de Cancérologie Gustave Roussy, F-94805, Villejuif, France; ^5^ EDST/PRASE, Rafic Harriri Campus, Faculté des Sciences, Université Libanaise, Beyrouth, Liban; ^6^ Laboratoire de Recherche sur la Croissance Cellulaire, la Réparation et la Régénération Tissulaires (CRRET), CNRS, F-94010, Créteil, France; ^7^ AP-HP, Hôpital H. Mondor, Département de pathologie, F-94000, Créteil, France; ^8^ Department of Medicine, Weill Cornell Medical College, New York, NY, 10065, USA; ^9^ AP-HP, Hôpital H. Mondor, Service d'urologie, F-94000, Créteil, France; ^10^ Mouse Cancer Genetics Program, Center for Cancer Research, Frederick National Laboratory for Cancer Research, Frederick, MD, 21702, USA

**Keywords:** prostate cancer, CRIPTO, mesenchymal-like cancer cells, EMT, FGFR, AKT

## Abstract

Members of the EGF-CFC (Cripto, FRL-1, Cryptic) protein family are increasingly recognized as key mediators of cell movement and cell differentiation during vertebrate embryogenesis. The founding member of this protein family, CRIPTO, is overexpressed in various human carcinomas. Yet, the biological role of CRIPTO in this setting remains unclear. Here, we find CRIPTO expression as especially high in a subgroup of primary prostate carcinomas with poorer outcome, wherein resides cancer cell clones with mesenchymal traits. Experimental studies in PCa models showed that one notable function of CRIPTO expression in prostate carcinoma cells may be to augment PI3K/AKT and FGFR1 signaling, which promotes epithelial-mesenchymal transition and sustains a mesenchymal state. In the observed signaling events, FGFR1 appears to function parallel to AKT, and the two pathways act cooperatively to enhance migratory, invasive and transformation properties specifically in the CRIPTO overexpressing cells. Collectively, these findings suggest a novel molecular network, involving CRIPTO, AKT, and FGFR signaling, in favor of the emergence of mesenchymal-like cancer cells during the development of aggressive prostate tumors.

## INTRODUCTION

Epithelial-mesenchymal transition (EMT) and its reverse process mesenchymal-epithelial transition (MET) are essential during different stages of embryonic development, tissue remodeling and cancer progression [1, 2]. During the EMT process, polarized epithelial cells loose their epithelial properties while gaining phenotypic properties of mesenchymal cells. These include a down-regulation of epithelial (E)-cadherin expression and tight junctional proteins such as occludins, increased expression levels of the mesenchymal cytoskeleton component, vimentin, and/or neuronal (N)-cadherin. In many types of tumors, EMT is believed to be an important step toward local invasion and subsequent tumor dissemination through lymphatic or hematogenous spread [2]. In addition, EMT may be important in the initiation or maintenance of a subpopulation of cancer stem cells [3]. However, the transient or metaplastic nature of EMT remains a major obstacle in conclusively demonstrating such processes in vivo either in primary or in secondary lesions. The task of identifying epithelial tumor cells that have undergone complete EMT is also complicated by the fact that they may resemble host tissue fibroblast or stromal cells. Additionally, numerous tumors may manifest EMT-like phenotypes through dedifferentiation rather than by a transdifferentiation [4]. Evidence is accumulating that EMT and MET are regulated by the activation of various signaling pathways and related growth factors such as FGFs, TGF-β, IGF, EGF and HGF, that are also known to perform essential biological roles during embryonic development and in normal adult tissues [1]. Prior work has also revealed that EMT may be influenced by several different embryonic signaling pathways [5, 6]. CRIPTO (also referred to as human Cripto-1, abbreviated **CR-1**) is the founding member of the EGF-CFC (Cripto, FRL-1, Cryptic) protein superfamily [7–9]. CR-1 and its orthologs are essential during embryogenesis acting as key regulators of embryonic stem cell differentiation [10, 11], mesendodermal fate, cell movement [12–15], and establishment of the anterior/posterior axis [16]. CR-1 functions as a membrane–associated protein to facilitate signaling by certain TGF-β subfamily of proteins such as Nodal, GDF3 and GDF1, while downregulating signaling by other ligands including activins and TGF-β1. CR-1 may also engage biological functions that are independent of Nodal or GDFs such by activating the SRC, MAPK or AKT signaling pathways [9] likely through its propensity to directly bind, or stimulate in trans, various transmembrane proteins such as ERBB4, GRP78, NOTCH, Glypican-1 and conceivably other as yet unidentified proteins. In addition, CR-1 is overexpressed in several different types of human carcinomas [9], but its biological role in these malignancies remains unclear. It has been suggested that CR-1 functions in conjunction with Nodal in cancer stem cell populations to promote tumorigenesis in melanoma and testicular tumors [17, 18]. Under some circumstances, CR-1 can promote cell proliferation, migration, invasion, or stimulate angiogenesis [19, 20]. On the other hand, it can promote apoptosis [21], or inhibit cell proliferation [22]. EMT-like events have also been observed in mammary glands derived from MMTV-CR-1 transgenic mice, or in various immortalized mammary epithelial cells forced to overexpress CR-1 (NOG-8, HC-11, NMuMG or MCF10A cells) [23–26]. A recent work provided some evidence that CR-1 promotes EMT in a specific population of non-small cell lung cancers mutated for EGFR [27]. Yet, it is unclear whether CR-1 overexpression can also contribute to EMT or EMT-like programs in other human malignancies [19, 25, 28, 29]

Recently, a number of studies have underscored the extraordinary plasticity of human prostate carcinoma (PCa) cells. Under various molecular and cellular perturbations, including aberrant activation of embryonic signaling pathways, these cells can transdifferentiate from epithelial to neuroendocrine-like cells [30], mesenchymal-like [31], or stem-like cells [32, 33]. To date, expression of CR-1 remains relatively uncharacterized in PCa; and albeit some expression has been detected in certain PCa cell lines, the functionality of this expression has yet to be established [34, 35] (Supplementary table S1). Only one study did assess CR-1 expression in a limited number (*n* = 9) of PCa specimens by immunohistochemistry and reported that CR-1 was absent in the malignant cells of these samples [36]. Here, we employed human PCa cells to explore further the possibility that CR-1 might contribute to EMT processes in human PCa, and define the possible mechanisms involved in this phenotypic transition. In addition, we aimed to define CR-1 expression pattern in a panel of normal, benign and malignant prostate tissues.

## RESULTS

### CRIPTO is overexpressed in a subset of primary human prostate adenocarcinomas

**Figure 1:**
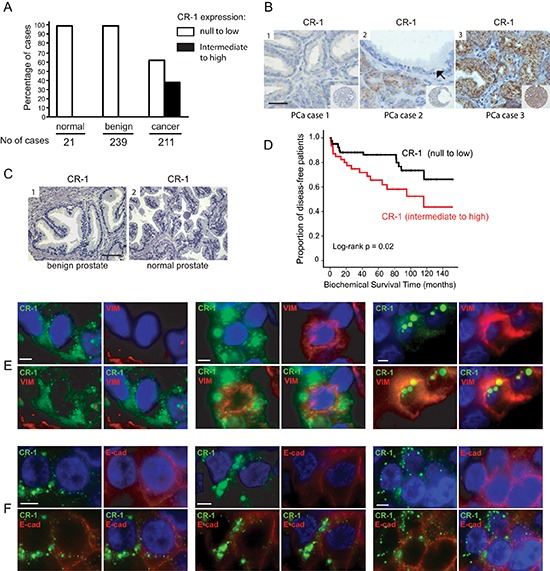
CRIPTO is expressed in a subset of aggressive human prostate cancers, and wherein resides a subpopulation of tumor cells with mesenchymal-like traits **(A)** Bar graph illustrating the repartition of CR-1 in human prostate carcinoma (cancer), normal prostate (normal) or benign prostate hyperplasia (BPH) cases. **(B)** Representative TMA elements immunostained with antibody to CR-1. Immunostaining shows absence of staining (1), intermediate staining in the cancer cells (2), and weak or marginal staining in the adjacent benign epithelial cells (*black arrows*), and strong granular staining (3) in PCa cells. The staining pattern is quite homogenous, granular, and in a perinuclear location. CR-1 does not appear to be expressed in the stroma. Scale bar, 50 μm. **(C)** Absence of immunostaining observed in BPH (1) and normal prostate (2) epithelial cells. Scale bar, 100 μm. **(D)** Kaplan–Meier estimate of the distribution of disease-free survival according to the CR-1 status in 136 patients. **(E)** Dual immunofluorescence on human PCa tissue sections identifies cancer cells coexpressing CR-1 and vimentin (middle and right). On the left, a case presenting with no co-expression pattern. **(F)** co-expression analysis of CR-1 and E-cadherin reveals partial loss of E-cadherin in neoplastic cells expressing significant amounts of CR-1 compared to adjacent cells. Scale bars, 10 μm.

We first assessed CR-1 mRNA expression by qRT-PCR in a series of human prostate tissue samples (33 cancerous and 7 normal) as well as in a panel of human normal and malignant prostate cell lines by qRT-PCR. CR-1 expression was especially high in a number of tumor specimens compared to non-malignant prostate specimens (Supplementary Figure S1A). Surprisingly, CR-1 mRNA transcripts were undetectable or poorly expressed, when compared to human tissues, in commonly used PCa cell lines and in several non-malignant immortalized prostate cell lines (Supplementary Figure S1B). Next, CR-1 expression was assessed immunohistochemically in pathological specimens consisting of 239 benign prostatic hyperplasia (BPH), and 211 PCa cases that were treated by surgical intervention. Significant CR-1 protein was detected in 80 of 211 PCas (37.9%) but was absent or marginally expressed in benign conditions such as BPH (Figure 1A–1C). The percentage of positively stained tumor cells was 67% on average and high levels of CR-1 in primary tumors was found to be associated with a higher risk of disease recurrence following surgery in univariate analyses (Figure 1D and Supplementary Table S2). The 3-year and 5-year recurrence-free survival was 71.8% and 65.6%, respectively, in patients with intermediate to high expression of CR-1 as compared to 88.2% and 86.3%, respectively, in patients with null to low expression of CR-1. No association was noted between CR-1 and conventional clinico-pathological parameters (Supplementary Table S2). Noticeably, multivariate analysis using a COX model, including Gleason grade, pT stage, lymph nodes and surgical margin status as post-operative co-variables, showed that CR-1 expression was an independent predictor of disease recurrence (*p* = 0.006; HR 3.01 [1.37–6.61])(Supplementary Table S3). In all, these data suggest CR-1 as a new biomarker with potential prognostic value for primary prostate cancer. We then investigated a possible link between CR-1 and EMT in human primary prostate tumors by examining expression of vimentin, a robust marker of mesenchymally-derived cells or cells undergoing an EMT. Dual immunofluorescence for CR-1 and vimentin, in most instances, showed the absence of vimentin in tumor cells while expression was found in the stromal contingent (Supplementary Figure S2A and S2B). In CR-1 immunopositive cases, a proportion of tumor cells did not show vimentin expression in which CR-1 expression was confined to the tumor epithelial cells (Figure 1E, left panel). Nevertheless, these tumors also seemed to harbor a subpopulation of neoplastic cells where CR-1 expression did coincide with vimentin expression (Figure 1E, middle and right panels). Moreover, we found several instances of neoplastic cells displaying especially high CR-1 expression along with reduced levels of expression of E-cadherin (Figure 1F). These observations suggested a link between CR-1 and acquisition of mesenchymal traits in PCa, and that at least a subpopulation of prostate neoplastic cells exhibit a significant mesenchymal-like phenotype.

### CRIPTO overexpression upregulates PI3K/AKT and ERK activities in 22Rv1 prostate cancer cells

**Figure 2:**
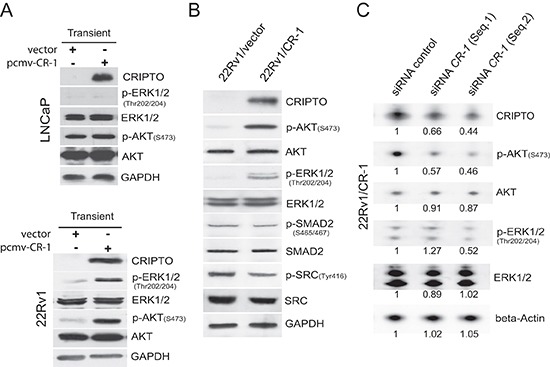
Phosphorylation of AKT and ERK1/2 is increased in human PCa 22Rv1 cells overexpressing CRIPTO **(A)** Western blot shows that 24h transfection with CR-1 increases phosphorylation of AKT and ERK1/2 in 22Rv1 cells but not in LNCaP cells. **(B)** The activation of AKT, ERK1/2, SRC, SMAD2 was compared in 22Rv1/vector and 22Rv1/CR-1 stable transfectants. **(C)** siRNA targeting of CR-1 in 22Rv1/CR-1 cells downregulates phospho-AKT and phospho-ERK1/2 at 72hr. The number below each lane is the quantified fold change (as evaluated by densitometry analysis) when compared with the first lane and when applicable after normalization to beta-actin (total ERK, AKT and CR-1), total ERK (p-ERK/1/2), or total AKT (p-AKT).

To mimic and study the situation of CR-1 overexpression that is found in some prostate tumors and tumor cells, we ectopically overexpressed CR-1 in two widely used PCa cell lines, LNCaP and 22Rv1 both of which possess an epithelial phenotype and extremely low levels of endogenous CR-1. LNCaP was originally derived from a lymph node metastatic lesion of human PCa [37], while 22Rv1 cells were originally derived from a primary site of an advanced PCa that was serially transplanted in nude mice before final isolation [38, 39]. Because earlier studies have found in various non-prostatic cancer models that CR-1 can signal via phosphatidylinositol 3-kinase (PI3K)/protein kinase B (AKT), and extracellular signal–regulated kinases 1/2 (ERK1/2) [9], we looked for perturbations of these kinases following transient overexpression of CR-1. Immunoblot analysis revealed elevated phosphorylation of AKT^(Ser473)^ and ERK1/2^(Thr202/Tyr204)^ in CR-1 transfected 22Rv1 cells but there was no change observed in activation of AKT or ERK1/2 in CR-1 transfected LNCaP cells (Figure 2A). To evaluate the effects of CR-1 overexpression in the long term, we then established stable transfectants from the pooled transiently transfected 22Rv1 cells. Stable overexpression of CR-1 in the cells (22Rv1/CR-1) was also associated with a significant up-regulation of phospho-AKT and phospho-ERK1/2 as compared to control transfectants (22Rv1/vector) (Figure 2B). By contrast, we could not find evidence of increased phosphorylation of SRC^(Tyr416)^, or of SMAD-2 at Serines 465/467 as typical readout for TGFbeta-related signal transduction including Nodal signaling (Figure 2B). In further experiments wherein cells were treated with the SRC inhibitor SU6656 or with ALK4/5/7 inhibitor SB431542, we could not find any inhibitory effects on the activation of ERK and AKT (data not shown). To exclude the possibility that aberrant activities of AKT and ERK1/2 resulted from non-specific events related to cell derivation, we then used different siRNAs to decrease human CR-1 expression in stably transfected 22Rv1/CR-1 cells. We found that a reduction of CR-1 levels was sufficient to attenuate phospho-AKT and phospho-ERK1/2 levels compared to control conditions (Figure 2C). Together, these results suggest that ectopic overexpression of CR-1 in 22Rv1 cells can increase transduction signals from AKT and ERK, with no apparent impact on other canonical and non-canonical TGF-beta superfamily smad2/3-dependent signaling pathways [40].

### A role for CRIPTO in epithelial to mesenchymal transition in prostate cancer cells

**Figure 3:**
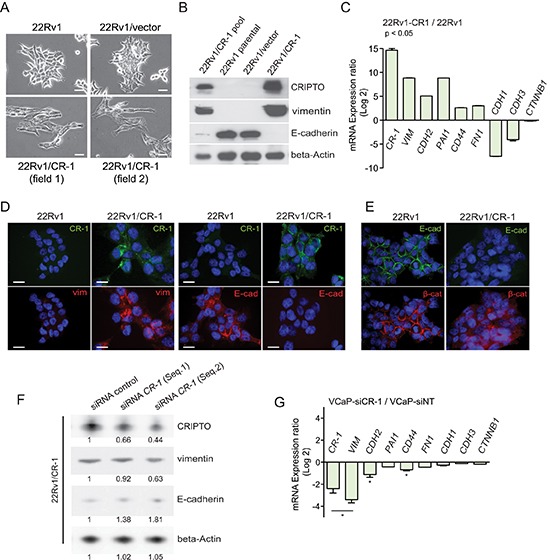
CRIPTO expression is positively associated with mesenchymal characteristics in cultures of PCa cells **(A)** Photomicrographs showing evidence of flattened mesenchymal-like morphology in CR-1 expressing 22Rv1 cells contrasting with more clustered epithelial-like organization of 22Rv1 or 22Rv1/vector cells. Scale bars, 100 μm. **(B)** Immunoblotting from cell lysates of 22Rv1 parental cells or its derivatives confirming the correlation between CR-1 and vimentin expression and E-cadherin loss. **(C)** qRT-PCR for mRNA expression levels of various EMT-related genes in 22Rv1/CR-1 compared to the 22Rv1 parental cells. Bars represent the means of two experiments ± SEM performed in triplicate. **(D)** Immunofluroescence analysis showing loss of E-cadherin and gain of vimentin in 22Rv1/CR-1 compared to 22Rv1 cells. **(E)** Immunofluroescence analysis showing relocalization of beta-catenin from adherens junctions of the membrane to cytoplasm in 22Rv1/CR-1 cells. Scale bars, 20 μm. **(F)** Immunoblots made against the indicated proteins from 22Rv1/CR-1 cells treated for 72h with control or two siRNAs targeting CR-1. The number below each lane is the quantified fold change (as evaluated by densitometry analysis) when compared with the first lane and relative to beta-actin (CR-1, E-Cadh and Vimentin). **(G)** qRT-PCR for mRNA expression levels of various EMT-related genes in CR-1 siRNA-treated VCaP cells relative to control Non-Targeting (NT) treated cells.

In 2D cultures, morphological changes were manifest with cells stably expressing CR-1 (22Rv1/CR-1) exhibiting a more mesenchymal-like morphology that was absent in the 22Rv1/vector or parental cells, suggestive of an EMT phenotype facilitated by CR-1 overexpression (Figure 3A). Western blot analyses indicated that CR-1 overexpressing 22Rv1 cells have reduced levels of E-cadherin and in contrast to vector-transfected cells, express significant amounts of vimentin (Figure 3B). A qRT-PCR analysis likewise demonstrated a clear overrepresentation of mesenchymal-associated markers *(VIM*, *CDH2* (N-cadherin), *CD44*, *PAI1* and *FN1)* in 22Rv1/CR-1 cells (Figure 3C). Immunofluorescence analysis confirmed increased vimentin expression and a loss in E-cadherin expression in 22Rv1/CR-1 cells (Figure 3D) accompanied by loss of β-catenin at cell-cell contacts (Figure 3E). Moreover, reduction of CR-1 levels with CR-1-targeted siRNAs led to a diminution in vimentin expression and an elevation of E-cadherin levels as assessed by Western blot analysis (Figure 3F). Taken together, these results indicate that *in vitro* CR-1 overexpression promotes EMT program in 22Rv1 PCa cells. To substantiate this finding, we sought to examine the effect of knocking-down CR-1 in VCaP cells which are known to exhibit a predominant epithelial phenotype with some mesenchymal attributes [41], while also expressing moderate levels of endogenous CR-1 mRNA (Supplementary Figure S1B). Consistent with the hypothesis that CR-1 sustains a mesenchymal phenotype in PCa cells, downregulation of CR-1 with CR-1-targeted siRNAs led to reduction of some mesenchymal-associated markers including *VIM*, *CDH2,* and *CD44* (Figure 3G)

### FGFR signaling contributes to the biological effects of CRIPTO on ERK1/2 activation in prostate cancer cells that is independent of AKT

**Figure 4:**
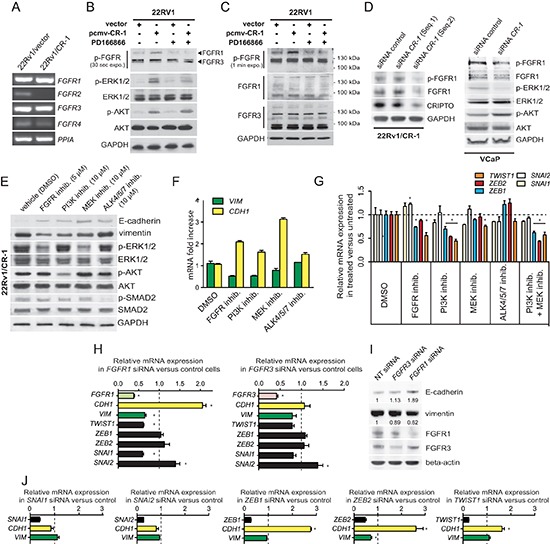
Mesenchymal differentiation elicited by CR-1 overexpression is mediated through parallel actions of AKT and FGFR signals and changes in expression of EMT-TF genes **(A)** RT-PCR analysis depicting the mRNA levels of the FGFR genes in 22Rv1/vector and 22Rv1/CR-1 cells. The housekeeping gene PPIA is used as an internal control **(B)** Immunoblots assessing the effect CR-1 cDNA transfection or control vector in 22Rv1, left untreated, or treated with FGFR inhib. (PD166866; 5 μmol/L). Phosphorylation of AKT, ERK1/2, and FGFR was analyzed. **(C)** The same samples were subjected to immunoblotting for FGFR1 and FGFR3 proteins. **(D)** Immunoblots made against the indicated proteins from 22Rv1/CR-1 and VCaP cells treated with control or two siRNAs targeting CR-1. **(E)** 22Rv1/CR-1 cells were treated with inhibitors to PI3K (LY294002, 10 μmol/L), MEK (U0126, 10 μmol/L), FGFR (PD166866, 5 μmol/L), ALK4/5/7 (SB 431542, 10 μmol/L) for 40h and lysates analyzed as above. **(F)** Parallel qRT-PCR analysis for *CDH1* (E-cadherin) and *VIM* (vimentin) mRNA levels. **(G)** qRT-PCR revealing perturbations of *ZEB1, ZEB*2 and *TWIST1* levels in inhibitor-treated 22Rv1/CR-1 cells. **(H)** qRT-PCR analysis for *FGFR1, FGFR3, CDH1, VIM*, and EMT-TFs following targeting of *FGFR1* or *FGFR3*. **(I)** Parallel WB analysis for E-cadherin and vimentin. **(J)** 22Rv1/CR-1 cells were treated with non-targeting siRNAs or siRNAs against *ZEB1, ZEB2, SNAI1, SNAI2*, or *TWIST1*, and mRNA levels of *CDH1, VIM*, and knockdown target genes were assessed by qRT-PCR. Error bars represent *n* = 3, mean ± sem.

Fibroblast growth factor (FGF) receptor 1 has been implicated in EMT in bladder cancer and PCa progression [42–44]. RT-PCR analysis indicated the presence of *FGFR1* and *FGFR3* mRNA as the most abundantly expressed FGFRs in 22Rv1 and 22Rv1/CR-1 cells (Figure 4A). In Xenopus, the ortholog of CR-1, FRL-1 (FGF receptor ligand 1) could potentially signal through the FGF receptor with respect to mesoderm induction in a manner that was independent of any detectable physical interactions between these two proteins [13]. Moreover, because FGFR receptors often operate through ERK and AKT, we reasoned that human CR-1 could indirectly influence FGFR signaling as a possible mechanism to increase ERK and/or AKT activation in the CR-1 transfected 22Rv1 cells. To test this, western blot analysis was made against phospho-(activated) FGFR from 22Rv1 cells transiently transfected with CR-1 or control vectors. The data revealed an increase in intensity of a 150/160-kDa band in CR-1 transfected cells in conjunction with an up-regulation of phospho-ERK and phospho-AKT levels while the 120/130-kDa phospho-FGFR bands remain unaffected (Figure 4B). Inspection of FGFR1 expression indicated a clear band at 150/160-kDa, but marginal expression at ~120/130-kDa, whereas FGFR3 appeared to be mainly represented by proteins with molecular weights of 130-kDa or smaller (Figure 4C). Thus in this setting, CR-1 may preferentially modulate FGFR1 activity but not FGFR3. Treatment of CR-1 transfected 22Rv1 cells with the FGFR inhibitor PD166866 (5μmol/L) attenuated the signal from the phospho-FGFR1 species as well as phospho-ERK levels but did not affect phospho-AKT levels (Figure 4B) suggesting regulation of ERK activity through FGFR1 signaling but little or marginal effects on AKT activity. Similar results were obtained when using TKI258 (1μmol/L) or PD166866 (5 μmol/L) as FGFR inhibitor in treating 22Rv1/CR-1 cells (Supplementary Figure S3).

In testing various FGFR1 expressing cell lines from different tissue types, we also found that CR-1 overexpression can have varied effects, from negative to positive effects on FGFR signaling suggesting that CR-1 may be a general modulator of this signaling pathway (Supplementary Figure S4). Moreover, reducing ectopic or endogenous expression of CR-1 in 22Rv1/CR-1 and VCaP cells resulted in a down-regulation of FGFR1 expression and/or activity in these cells consistent with the notion that CR-1 can positively regulate FGFR1 signaling in PCa cells (Figure 4D). We noted no significant effects on phospho-AKT levels while phospho-ERK was reduced in CR-1-siRNA treated VCaP cells again suggesting a lack of effect of FGFR signaling on AKT activation in PCa cells. Altogether, these observations suggest that FGFR activity can be promoted by CR-1 expression thereby influencing ERK activity but with no apparent direct effects on AKT activity.

### CRIPTO mediated EMT is compromised by targeted inhibition of MAPK, AKT or FGFR activity

Next, we sought to assess the effects pharmacological blockade of PI3K and MAPK/ERK kinase (MEK), two upstream activators of AKT and ERK1/2, respectively. Inhibition of PI3K using LY294002 (10 μmol/L) or of MEK using U0126 (10 μmol/L) resulted in a decrease of phospho-AKT and phospho-ERK levels, respectively, an increase in E-cadherin levels, and a reduction in vimentin expression (Figure 4E). In addition, cells that were exposed to the FGFR inhibitor PD166866 (5 μmol/L) continued to express substantial levels of phospho-AKT but had reduced levels of phosphorylated ERK1/2 in conjunction with an up-regulation of E-cadherin expression and a down-regulation of vimentin expression. Interestingly, both FGFR and PI3K inhibitors attenuated the signal from the phospho-FGFR1 suggesting regulation of FGFR1 by PI3K/AKT through direct or indirect mechanisms (Supplementary Figure S5). As CR-1 is a known co-receptor for the TGF-β protein Nodal, pharmacological inhibition of Nodal receptors, ALK4/7 was also investigated using SB432541. Treatment of the 22Rv1/CR-1 cultures with SB432541 had no noticeable effect on phosphorylation of ERK or AKT, nor did this significantly affect expression of vimentin (Fig. 4E), although we noted a small increase in E-cadherin expression. These results were further confirmed at the mRNA level (Figure 4F). Collectively, this data indicates that 22Rv1/CR-1 cells have enhanced AKT and ERK/MAPK activities by upstream stimulation of the FGFR1, and PI3-kinase activities, and that pharmacological inhibition of these pathways impedes CR-1-mediated effects in PCa cells.

Several E-box binding transcription factors (TFs) known to regulate EMT [1] are up-regulated in the 22Rv1/CR-1 cells as compared to control cells (Supplementary Figures S6A and S6B). This includes *ZEB1* (11-fold), *ZEB2* (15-fold), *SNAI1/*SNAIL (4-fold), *SNAI2/*SLUG (12-fold)*, TWIST1* (2.5-fold). To further define the molecular pathways that operate downstream of the FGFR, PI3K/AKT and ERK activation, we examined expression levels of these EMT-TFs in the inhibitor-treated 22Rv1/CR-1 cells. PI3K inhibition alone or in combination with MEK inhibition, reduced *ZEB2* and *TWIST1* mRNA levels by approximately 2-fold and *ZEB1* level to a lower extent (Figure 4G). *SNAI1* and *SNAI2* levels remained relatively unchanged under these same conditions. The TGF-β. ALK4/5/7 inhibitor had no significant effects on expression of any of these genes (Figure 4G). FGFR inhibition and MEK inhibition decreased *TWIST1* mRNA level but had little to no effect on *ZEB1* and *ZEB2* mRNA levels. To further test the contribution of FGFR1 and FGFR3 receptors, we knocked-down *FGFR1* or *FGFR3* expression in the cells and examined expression levels of EMT-TFs*, CDH1 (*E-cadherin) and *vimentin*. *FGFR1* knockdown was associated with a down-regulation of *TWIST1* and *SNAI1* expression as well as an upregulation of E-cadherin expression and a decrease in vimentin expression (Figure 4H–4I). By contrast, targeting *FGFR3* had little to no effect on *TWIST1*, *SNAI1,* E-cadherin or vimentin expression in the 22Rv1/CR-1 cells suggesting that FGFR1 has an important role in enforcing the mesenchymal phenotype in CR-1 overexpressing PCa cells (Figure 4H–4I).

In an attempt to confirm the contribution of the respective EMT-TFs in the phenotype of the cells, we performed transient knockdown of *SNAI1*, *SNAI2*, *TWIST1*, *ZEB1* or *ZEB2,* and evaluated vimentin and E-cadherin (*CDH1)* within 72h post-transfection. In siRNA-transfected cells, *ZEB2* silencing led to significant reduction in vimentin expression levels, and targeting *ZEB2* and *TWIST1* was consistently accompanied by an increase in E-cadherin at the RNA and protein levels that could not be recapitulated by depletion of *SNAI1* or *SNAI2* (Figure 4J and supplementary Figure S7). Collectively, these results support a model in which ZEB2 and TWIST1 are predominantly involved in regulating the mesenchymal phenotype in the CR-1 overexpressing PCa cells.

### CRIPTO/AKT/FGFR stimulates cell growth, migration and invasion that are compromised by pharmacological inhibition of ERK, AKT or FGFR

**Figure 5:**
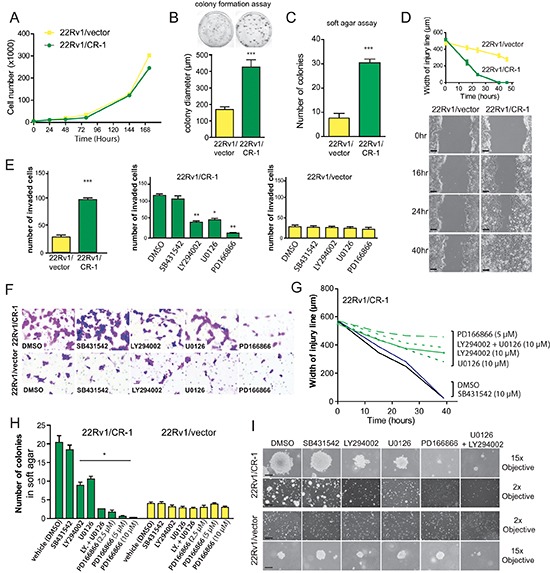
CRIPTO overexpression is associated with increased malignant properties of prostate cancer cells **(A)** Anchorage-dependent growth rates of 22Rv1/vector and 22Rv1/CR-1 cells over a 7-day period in standard medium. **(B)** 22Rv1/vector and 22Rv1/CR-1 cells were assayed for colony formation in monolayer cultures **(C)** Colony formation ability of 22Rv1/CR-1 cells compared to 22Rv1/vector cells in soft agar. **(D)** The two lines were analyzed for migratory by photography 0, 24 or 40 hours after wounding; Scale bars, 200 μm. Width of the injury line at the different time points is depicted. **(E)** Invasion of the two lines as assessed by Boyden chamber assay under untreated or treated conditions. **(F)** Representative images of invaded cells (microscopic fields at 20x objective magnification). **(G)** Width of injury line from wounding-healing assays in untreated or treated cells. **(H)** The 22Rv1/CR-1 and 22Rv1/vector cells were grown in soft agar for three days and then treated with the indicated inhibitors for an additional 11 days. The number of colonies presented is the mean of colony counts in ten 100x microscopic fields from three wells. **(I)** Representative photomicrographs from the previous experiment; Scale bars, 150 μm, 1000 μm. All assays were performed in triplicate. Bars; Means, Error bars, ± sem ; *** *p* < 0.001. ** *p* < 0,01.* *p* < 0,05.

We then investigated the behavior of 22Rv1/vector and 22Rv1/CR-1 cells in terms of cell migration, invasion and growth in 2D or 3D cultures. The two PCa lines had comparable growth rate in normal monolayer cultures (Figure 5A). When seeded at low density, the size of colonies derived from the 22Rv1/CR-1 cells were substantially larger than those generated from 22Rv1/vector cells (Figure 5B). In addition, the 22Rv1/CR-1 cells were able to form more colonies when grown in soft agar (Figure 5C). Migration and invasive assays showed that the 22Rv1/CR-1 cells were much more motile (Figure 5D) and invasive (Figure 5E) than control-transfected cells within 48hrs. Moreover, treatment with the FGFR, PI3K or MEK inhibitors substantially reduced invasion, migration and colony formation capacities of 22Rv1/CR-1 as opposed to minor effects in the 22Rv1/vector control cells (Figs. 5F–5J). These data further implicate FGFR, ERK1/2, and PI3K/AKT components as key signaling molecules linked to CR-1-mediated biological effects.

## DISCUSSION

EMT has been observed in a number of different types of carcinoma cells [1, 4]. Despite an extensive amount of experimental data, the relevance of EMT is still disputed by the fact that cancer cells undergoing EMT are hardly detectable in biological fluids or in tissue specimens. To our knowledge, this report represents first demonstration for the existence of a population of CR-1 expressing human PCa cells that manifests mesenchymal-like characteristics within primary tumors. We conclude that this CR-1 high expressing population may represent at least one subtype of PCa mesenchymal-like cells.

**Figure 6:**
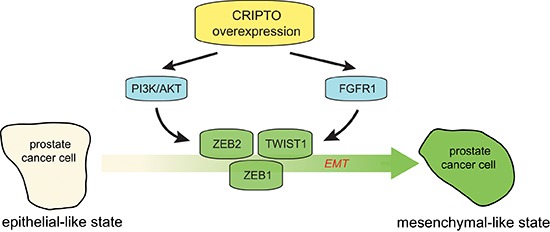
Working model of the regulatory role of CRIPTO in regulating epithelial-mesenchymal transition in human prostate cancer cells Aberrant CR-1 expression can increase the activation of AKT and FGFR1, leading to deregulation of prostate epithelial differentiation towards a mesenchymal state that is coordinated by ZEB1, ZEB2 and TWIST1.

Whereas we interpret our findings as supportive of the idea that CR-1 mediates EMT process within some primary tumors, it remains unproven whether CR-1 can play a similar role in a metastatic context. Future work should focus on determining if CR-1 directly participates in metastatic progression, or if it predisposes to metastatic spread and the development of pertinent *in vivo* models is now warranted. By analogy to what occurs during development, it is conceivable that there may be multiple rounds of EMT and MET in the life of a cancer cell (primary versus metastatic sites) and that are potentially controlled by signals which are diverse and produced by different microenvironments [2, 45]. Presently, we speculate that CR-1 might initiate EMT early in the course of cancer progression. This may reflect a scenario also seen in developing embryos where mouse Cr-1, or zebrafih cripto, *oep,* likely participate in early EMT processes allowing ectodermal epiblast cells to migrate through the primitive streak during gastrulation [12, 14, 16].

With respect to the mechanisms involved, we found that overexpression of CR-1 in 22Rv1 PCa cells enhanced the activity of AKT, and ERK1/2 downstream of the FGFR1. We identified CR-1 as a positive regulator of FGFR1 signaling and this signaling seems to operate in the CR-1 overexpressing cells to support their mesenchymal state with a reduced role for FGFR3. Consistent with our data, recent work using mouse embryos showed that conditional inactivation of mouse Cripto-1 can lead to perturbation of Fgfr1 expression along with defects in mesoendoderm development [15]. In bladder cancer cells, FGFR3 expression directly correlates with an epithelial-like state whereas FGFR1 expression correlates with a more mesenchymal-like state [44].

Another intriguing aspect emerging from our study is the possibility that some mesenchymal-like cancer cells may be using PI3K/AKT-signaling to regulate FGFR signaling, an effect evocative of recent studies which established the role of PI3K/AKT in upregulating the expression and/or activity of certain RTKs such as ERBB3 [46, 47]. This could underlie a complex regulatory network in place between FGFR1, FGFR3, or some RTK receptors, and PI3K/AKT with impact on controlling the mesenchymal state in the cells.

Recent studies also highlight the potential roles of N-cadherin and receptor tyrosine kinases (RTKs) in mediating EMT and FGFR functions [48] and such regulatory events have yet to be studied in the context of PCa. In contrast to that found for ERK, the ability of CR-1 to activate AKT was not exerted through FGFR signaling. Our observation that SRC activity was not significantly affected by CR-1 overexpression also contrasts with prior work suggesting that increased SRC activation is necessary for cripto-dependent activation of ERK and AKT [9, 25, 27]. This suggests that there may be context-dependent regulation of different intracellular effector pathways by CR-1. The limited role of Src in this setting could be explained by important perturbations occurring at cell-cell contacts. Moreover, our unpublished observations seem to indicate that GRP78 (*HSPA5*) which is a known binding partner of CR-1 that can signal through SRC has limited functions in the current model [49]. *ERBB4* was downregulated in CR-1 overexpressing cells and Glypican-1 (*GPC1*), a putative binding partner of CR-1, was found to be marginally expressed in the PCa cell lines [50, 51]. This also argues for the existence of context-dependent activation of SRC, ERK and AKT that might explain, some contradictory reports that have demonstrated differential effects of CR-1 in modulating cell proliferation, survival/apoptosis or transformation properties [19, 21, 22, 49, 52].

Given the growing interest in targeting FGFR and PI3K/AKT as therapeutic target in cancer [53, 54], future studies should explore these questions and the potential relationships between different signaling pathways during cancer progression.

Another aspect emerging from this study is that perturbation of oncogenic signaling pathways (PI3K/AKT, FGFR/MAPK) which are activated downstream of CR-1 can impair the EMT program and the expression of EMT-TFs such as *TWIST1*, *ZEB1* and *ZEB2.* Inhibition of the expression of these EMT-TFs, can disrupt EMT in PCa cells. Hence we suggest that one notable function of CR-1 is to augment AKT as well as FGFR/ERK signaling which can promote EMT and sustain a mesenchymal-like phenotype by regulating ZEB1, ZEB2, and TWIST1 expression. This data continues to support a role for EMT in prostate cancer progression [31] as well as the pivotal roles of TWIST1 and ZEB factors in cancer cells from various tissues [6, 55–57]. Certainly one confounding aspect in this model is the seeming less prominent role for *SNAI1* and *SNAI2*. Nonetheless, we envision these two variables may be at play in the potential cross-talk involving FGFRs and PI3K/AKT. Another potential explanation comes from the complex interactions that may occur in our model, between CR-1 and other components of the TGF-beta signaling pathway or molecules that interfere with TGF-beta signaling [40, 49, 58, 59]. Pharmacological inhibition of the receptors for Nodal signaling, ALK4/7, had limited effects in our experimental conditions. Our preliminary investigations also indicate that Nodal mRNA level is relatively low in CR-1 expressing VCaP and 22Rv1/CR-1 cell lines (supplementary Figure S8). Nevertheless, we should consider the possibility that Nodal might function in conjunction with CR-1 at a specific time or in certain tumor subpopulations that may have cancer stem-like properties and that have the capacity to promote tumor development [17, 18, 60, 61]. Clearly this is an area in which more research is required.

One important challenge in the area of PCa research is to develop novel methodologies and biomarkers that can better assess aggressiveness of prostate tumors [62]. This endeavor could help guide therapy for individual patients, and avoid unnecessary risk for those that do not need treatment. In this study, we identified CR-1 as a novel candidate marker to predict disease recurrence in patients primarily managed by surgery. Further evaluation is warranted and should interrogate larger and independent cohorts. In addition, because PSA biochemical recurrence does not always appear to be an accurate predictor of cancer specific death in men with PCa [63], the impact of CR-1 expression on overall survival has yet to be addressed. At least, this first study suggests that CR-1 may be useful in addition to current prognostic tools for predicting local treatment outcome. In other human malignancies, with the possible exception of breast cancer [64], it is still unclear as to whether CR-1 may have prognostic significance. Moreover, the mechanisms and factors that contribute to CR-1 overexpression have yet to be identified. Clearly, further research regarding the selective expression of CR-1 in certain malignancies may be valuable for unraveling its multifaceted role in development and in cancer.

## METHODS

### Cell culture and reagents

22Rv1 and VCaP cell lines were obtained from the American Type Culture Collection (Manassas, VA) and authenticated at this site. Cells were maintained in RPMI-1640 medium supplemented with 10% FBS. 22Rv1 stable transfectants were generated using the CR-1 cDNA cloned into the p3XFLAG-Myc-CMV-25 expression vector (Sigma Aldrich, St Louis, MO), or empty vector, and transfection by Lipofectamine 2000 (Life Technologies, Grand Island, NY) followed by G418 selection at 400 μg/mL for three to four weeks. LY294002 and U0126 were purchased from LC Laboratories (Woburn, MA), PD166866 and SB431542 were from Sigma Aldrich.

### RNA preparation, cDNA synthesis and Quantitative Real-Time PCR

Total RNA extraction was performed using RNeasy Mini Kit from Qiagen (Valencia, CA) and subjected to DNase treatment (DNA-free kit; Life technologies). Reverse transcription was performed using Maxima™ Reverse Transcriptase (Thermo Scientific, Waltham, MA) followed by *q*PCR using iTaq SYBR Green supermix (Bio-Rad, Richmond, CA) on an Applied Biosystems 7900 Real Time PCR system (Applied Biosystems, Foster City, CA). Sequences of the oligonucleotide primers are provided in Supplementary Table S4.

### Protein preparation and western blot analysis

Protein lysates were prepared as previously described in RIPA [65]. Western blot analysis was performed with antibodies specific for beta-actin, Flag (Sigma Aldrich), GAPDH (Life technologies), CR-1, E-cadherin (Epitomics), vimentin (Dako, Trappes, France), p-SRC, SRC, p-SMAD2, p-ERK1/2, ERK1/2, p-FGFR1-4, p-AKT, AKT (Cell Signaling), FGFR3, FGFR1, SMAD2 (Santa Cruz, CA, USA).

### *In vitro* assays

The following siRNAs from Thermo Scientific were used in this study and transfected with lipofectamine 2000: ON-TARGET plus Non-Targeting Pool (D-001810), ON-TARGET plus to *SNAI1* (L-010847), *SNAI2* (L-017386), *ZEB1* (L-006564), *ZEB2* (L-006914), *TWIST1* (L-006434), *FGFR1* (L-003131), *FGFR3* (L-003133), or individual siRNAs composing SMARTpool ON-TARGET plus to *TDGF1* (L-004832).

Soft agar assays were performed as previously described [65]. For Colony formation assays, 500 cells were seeded at low confluence in 60-mm plastic Petri dishes, and grown in standard medium. After 14 days, colonies were stained with crystal violet, counted, and the diameters of the colonies measured. For wound closure assays, a wound (~500 μm) was scratched into confluent cultures. Wound regions with an identical width were marked, and wound closure was measured using photographs of 10 randomly selected wound areas at the time points indicated. Invasion assays were performed as previously [65] with small modifications. 9 × 10^4^ cells/well that were resuspended in 0.5 mL of RPMI FBS free medium were placed into Matrigel-coated Transwell insert containing 8-μm filters with the bottom well filled with RPMI 10% FBS. When indicated, inhibitors or vehicle were added to the serum containing media. After 48 h, the filters were fixed and stained with Crystal Violet 0.5% for 30 min, and invaded cells quantified.

### Patient samples

The prostate samples have been collected as part of an Institutional Review board- approved protocol at Henri Mondor Hospital (France). The cohort consisted of 256 patients who had undergone radical prostatectomy for clinically localized prostate cancer, and 239 benign prostatic hyperplasia (BPH) cases from patients that were surgically treated for BPH. The study also included a few specimens derived from normal prostates of young donors. Specimens were reviewed by a referee genitourinary pathologist (Y.A).

### Immunohistochemical analyses

Paraffin-embedded tissues were sectioned at 5 μm thickness and evaluated by immunohistochemistry (IHC) on tissue microarrays. After antigen retrieval using microwave heating, CR-1 protein expression was assessed following ABC immunohistochemistry using a human CR-1 mouse monoclonal antibody (R&D Systems, Minneapolis, MN) as previously described [66], omitting the proteinase K treatment. Specificity of the antibodies in prostate tissues was verified in immunocompetitive experiments (Supplementary Figure S9). Granular staining was considered and scored as null (0), weak (1), moderate (2) or strong (3). At least two interpretable cores were required to include a case for analysis. The percentage of positive tumor cells in each core was estimated and a case considered with intermediate to high expression of CR-1 when greater than 25% of the epithelial cells displayed positive immunoreactivity (score 1–3). We considered the other cases as null to low expression. For dual immunofluorescence staining, samples were processed as above but using as secondary antibodies, anti–mouse Alexa Fluor 488 (Life technologies) and biotinylated anti-rabbit antibodies (Jackson ImmunoResearch, Suffolk, UK) with subsequent incubation with Streptavidin-Fluoprobes 647H (Interchim, Montluçon, France). Rabbit primary antibodies used in co-expression studies were from Epitomics (Burlingame, CA; anti-E-cadherin, anti-vimentin). Slides were mounted using Vectashield mounting medium containing DAPI (Vector Laboratories, Burlingame, CA, USA) for microscopic inspection. Microscopic images were obtained under a 60x oil immersion objective using a Zeiss Axioplan2 microscope (Carl Zeiss, Le Pecq, France)

### Statistical analysis

Statistical tests used a two-tailed α = 0.05 level of significance and were performed using SPSS 13.0 for Windows. For *in vitro* studies, comparisons between groups were performed using the Wilcoxon or Mann-Whitney tests. For *in situ* studies, the chi-square test or Fisher's exact test were applied to assess associations between groups and clinicopathological variables. Recurrence-free survival curves were generated by the Kaplan-Meier method and compared using the log-rank test. The day of surgery represent the starting point of analysis while biochemical recurrence was defined as first detectable elevation of PSA above 0.20 ng/mL. Multivariate analysis was carried out using the Cox procedure.

## SUPPLEMENTARY FIGURES AND TABLES


