# Erratum: CRIPTO overexpression promotes mesenchymal differentiation in prostate carcinoma cells through parallel regulation of AKT and FGFR activities

**DOI:** 10.18632/oncotarget.27038

**Published:** 2019-06-25

**Authors:** Stéphane Terry, Ihsan Y. El-Sayed, Damien Destouches, Pascale Maillé, Nathalie Nicolaiew, Guillaume Ploussard, Fannie Semprez, Cynthia Pimpie, Himisha Beltran, Arturo Londono-Vallejo, Yves Allory, Alexandre de la Taille, David S. Salomon, Francis Vacherot

**Affiliations:** ^1^Inserm, U955, Equipe 7, Créteil, France; ^2^Université Paris-Est, UMR_S955, UPEC, F-94000, Créteil, France; ^3^Institut Curie, Centre de Recherche, CNRS UMR 3244, Paris, F-75248, France; ^4^Inserm, U753, Institut de Cancérologie Gustave Roussy, F-94805, Villejuif, France; ^5^EDST/PRASE, Rafic Harriri Campus, Faculté des Sciences, Université Libanaise, Beyrouth, Liban; ^6^Laboratoire de Recherche sur la Croissance Cellulaire, la Réparation et la Régénération Tissulaires (CRRET), CNRS, F-94010, Créteil, France; ^7^AP-HP, Hôpital H. Mondor, Département de pathologie, F-94000, Créteil, France; ^8^Department of Medicine, Weill Cornell Medical College, New York, NY, 10065, USA; ^9^AP-HP, Hôpital H. Mondor, Service d’urologie, F-94000, Créteil, France; ^10^Mouse Cancer Genetics Program, Center for Cancer Research, Frederick National Laboratory for Cancer Research, Frederick, MD, 21702, USA

**This article has been corrected:** During the assembly of the Figure 5 panel I, low magnification images (2x objective) from the same condition SB431542 were inadvertently used for both the vehicle DMSO and SB431432 treated 22Rv1/CR-1 cells. Similarly, the low magnification image (2x objective) from LY294002 treated 22rv1/vector was inadvertently used for both LY294002 and U0126 treated cells. The corrected Figure 5 is shown below. The authors declare that these corrections do not change the results or conclusions of this paper.

Original article: Oncotarget. 2015; 6:11994–12008. 11994-12008 . https://doi.org/10.18632/oncotarget.2740

**Figure 5:**
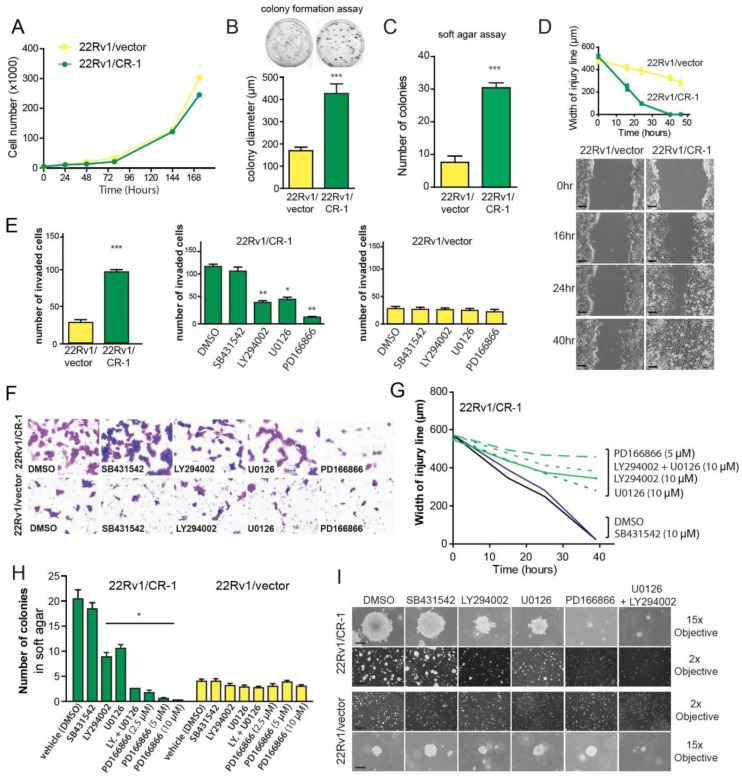
CRIPTO overexpression is associated with increased malignant properties of prostate cancer cells. (**A**) Anchorage-dependent growth rates of 22Rv1/vector and 22Rv1/CR-1 cells over a 7-day period in standard medium. (**B**) 22Rv1/vector and 22Rv1/CR-1 cells were assayed for colony formation in monolayer cultures (**C**) Colony formation ability of 22Rv1/CR-1 cells compared to 22Rv1/vector cells in soft agar. (**D**) The two lines were analyzed for migratory by photography 0, 24 or 40 hours after wounding; Scale bars, 200 μm. Width of the injury line at the different time points is depicted. (**E**) Invasion of the two lines as assessed by Boyden chamber assay under untreated or treated conditions. (**F**) Representative images of invaded cells (microscopic fields at 20x objective magnification). (**G**) Width of injury line from wounding-healing assays in untreated or treated cells. (**H**) The 22Rv1/CR-1 and 22Rv1/vector cells were grown in soft agar for three days and then treated with the indicated inhibitors for an additional 11 days. The number of colonies presented is the mean of colony counts in ten 100x microscopic fields from three wells. (**I**) Representative photomicrographs from the previous experiment; Scale bars, 150 μm, 1000 μm. All assays were performed in triplicate. Bars; Means, Error bars, ± sem; *** *p* < 0.001. ** *p* < 0,01.* *p* < 0,05.

