# Supplementary material for: CRIPTO overexpression promotes mesenchymal differentiation in prostate carcinoma cells through parallel regulation of AKT and FGFR activities
Source: Oncotarget. 2015 Jan 22;6(14):11994–2008. doi: 10.18632/oncotarget.2740 (PMC4494918; doi:10.18632/oncotarget.2740)
Supplement: Supplementary file 1 [file oncotarget-06-11994-s001.pdf]

SUPPLEMENTARY FIGURES AND TABLES

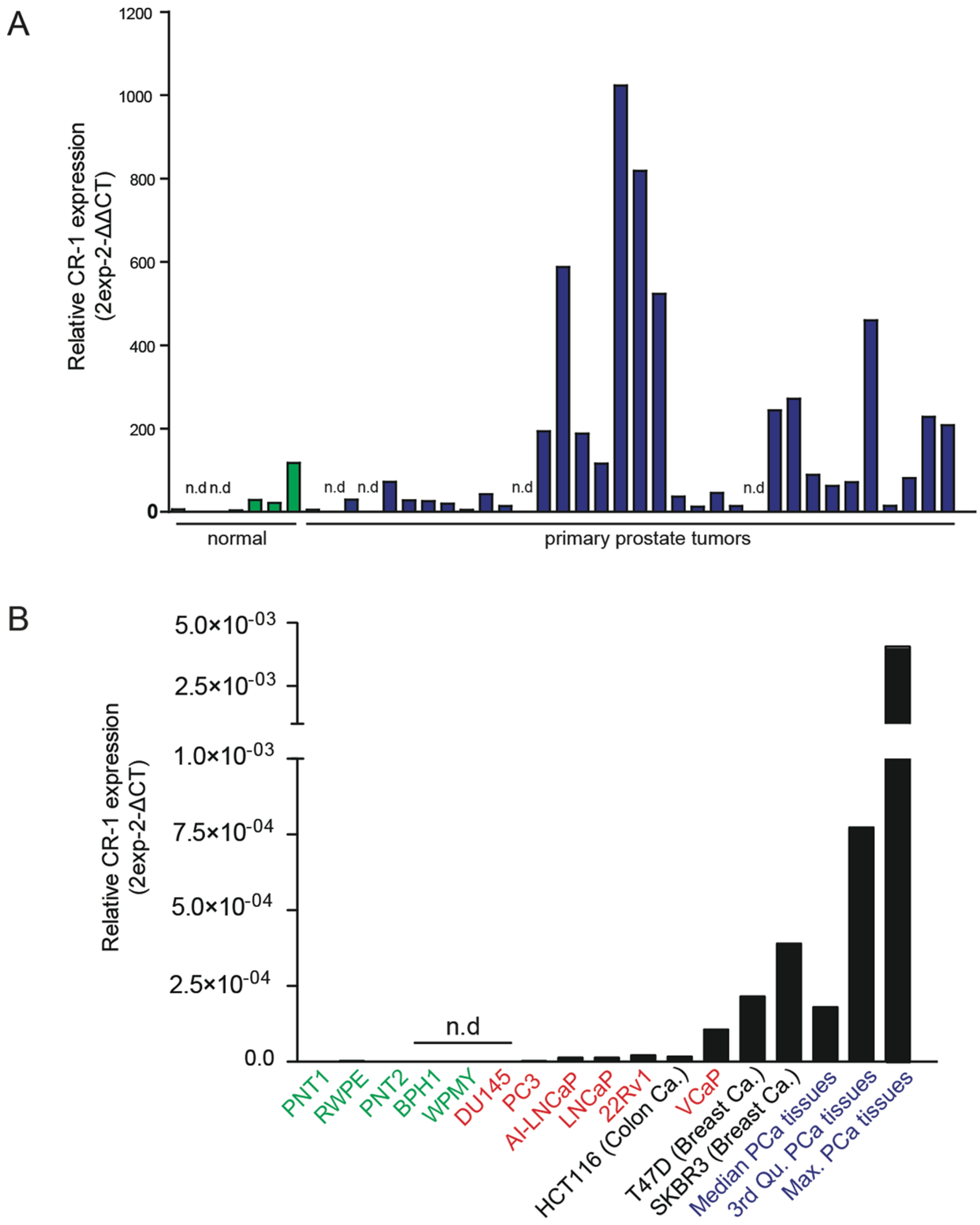

Supplementary Figure S1: *CR-1* transcript expression in prostate tissues and cell lines. (A) qRT-PCR analysis showing expression of *CR-1* mRNA in 33 specimens of primary prostate tumors and 7 adjacent normal tissues (normal). Normalized expression levels (against *RPLP0* and *PPIA)* are indicated as fold increase relative to expression levels found in the immortalized prostatic epithelial cell lines PNT1A and RWPE. **(B)** A set of immortalized prostate cell lines (green), prostate cancer cell lines (red) was examined for CR-1 mRNA expression. Expression levels found in PCa tissues are included for comparison (blue) as well as well-known non-prostatic cell lines HCT116, T47D and SKBR3. SKBR3 is known to express modest levels of CR-1 [6]

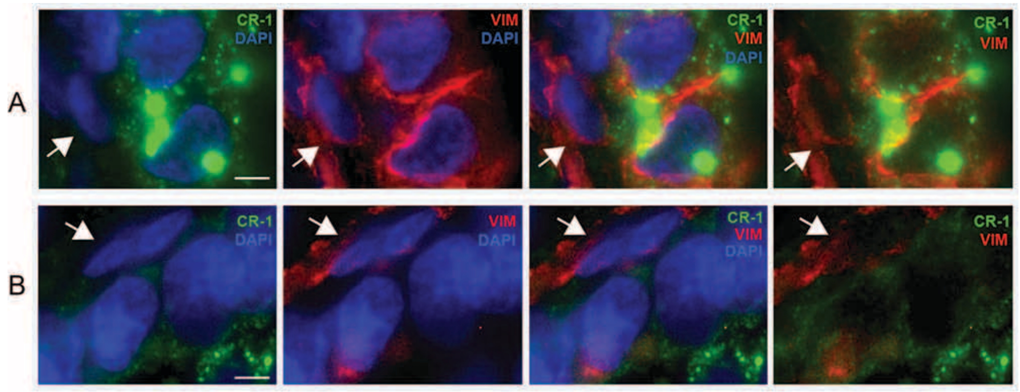

Supplementary Figure S2: Absence of CR-1 in stromal cells adjacent to neoplastic cells in human prostate cancer. Immunofluorescence analysis of CR-1 (Green) and vimentin (Red) in PCa tissue sections. Note the non-tumoral stromal cells characterized by small nuclei showing positivity for vimentin but negativity for CR-1 (**A** and **B**, white arrows). In **(A)** vimentin is expressed both in stromal and neoplastic cells, and simultaneous expression of CR-1 and vimentin is only seen in neoplastic cells. In **(B)** CR-1 expression is again restricted to neoplastic cells, while vimentin expression is mainly confined to two juxtaposed stromal cells (white arrow).

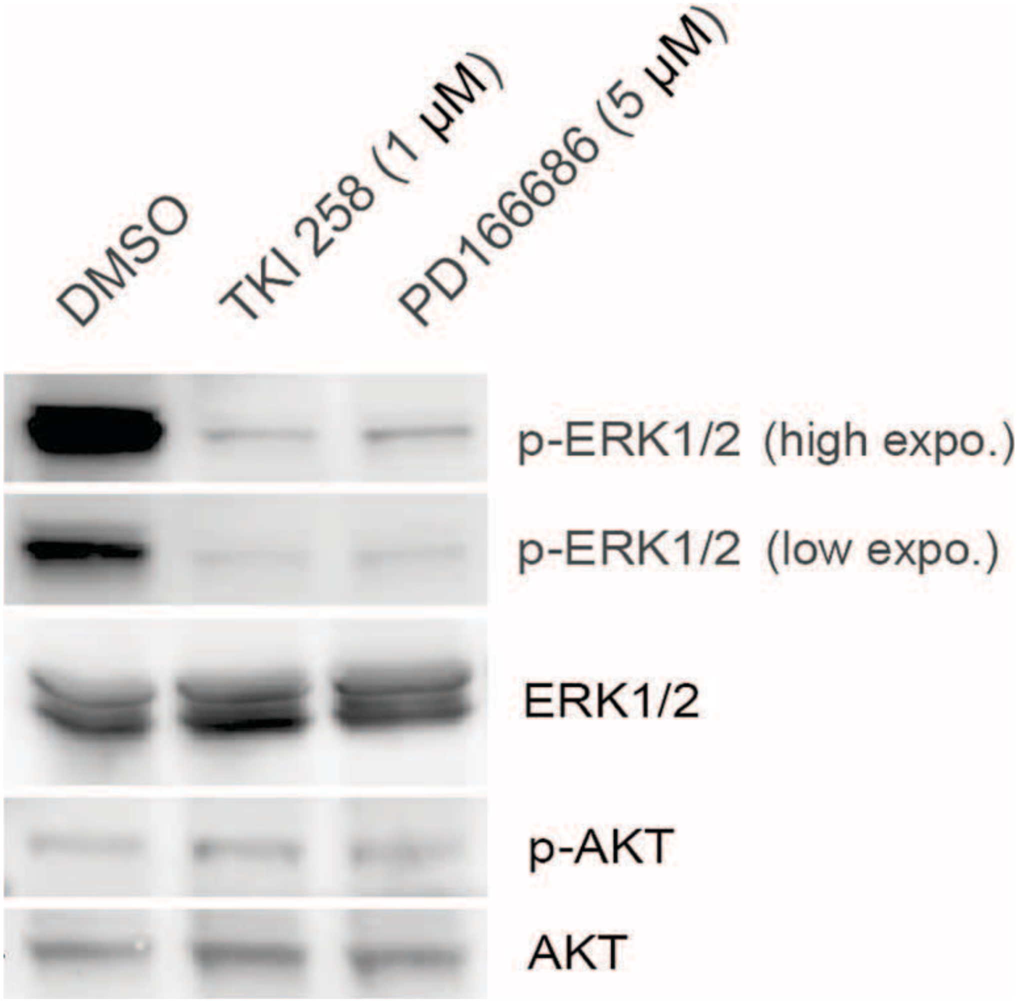

Supplementary Figure S3: Pharmacological inhibition of FGFR reduced ERK phosphorylation but not AKT phosphorylation in 22Rv1/CR-1 cells. 22Rv1/CR-1 cells were treated with inhibitors to FGFR (PD166866, 5 μmol/L or TKI258, 1 μmol/L) for 40h, and lysates analyzed for total and phosphorylated ERK and AKT proteins.

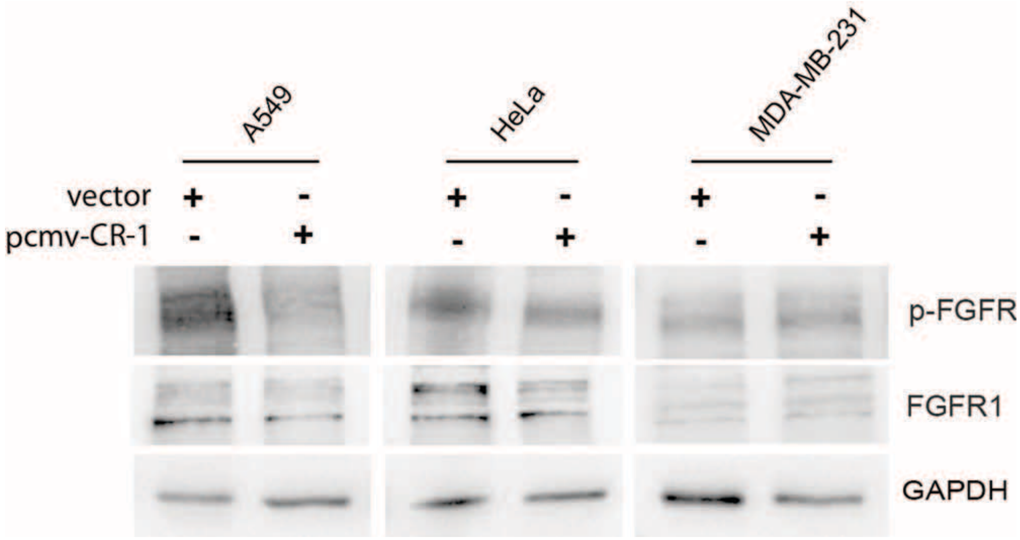

Supplementary Figure S4: Effect of ectopic expression of CR-1 on phospho-FGFR and FGFR1 in various cell lines. Breast (MDA-MB-231), lung (A549), cervical (Hela) carcinoma cell lines were transfected with either empty or CR-1 expression vectors and harvested at 48h post-transfection. Immunoblots for phospho-FGFR, FGFR1 and GAPDH used as loading control.

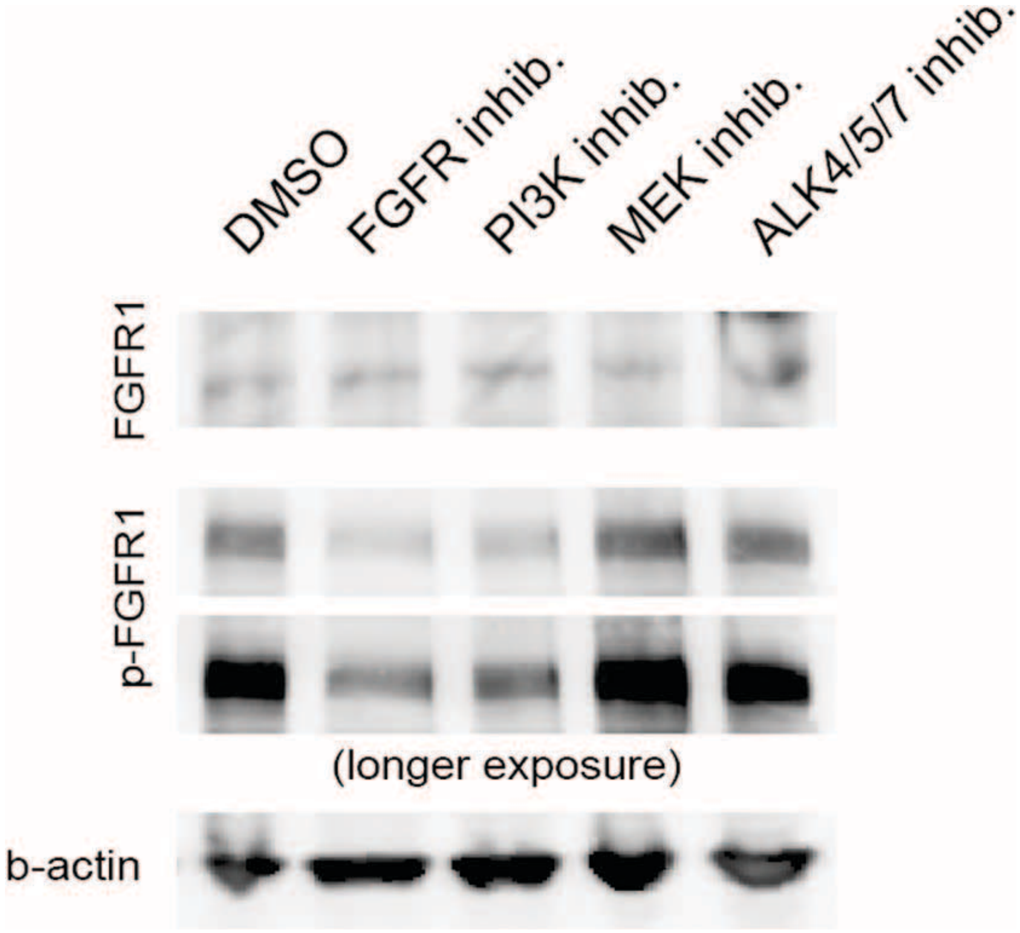

Supplementary Figure S5: FGFR as well as PI3K inhibition inhibited FGFR1 activity in 22Rv1/CR-1 cells. Immunoblots made against the phospho- and total FGFR1 forms from 22Rv1/CR-1 cells treated with inhibitors to PI3K (LY294002, 10 µmol/L), MEK (U0126, 10 µmol/L), FGFR (PD166866, 5 µmol/L), or ALK4/5/7 (SB 431542, 10 µmol/L) for 40h

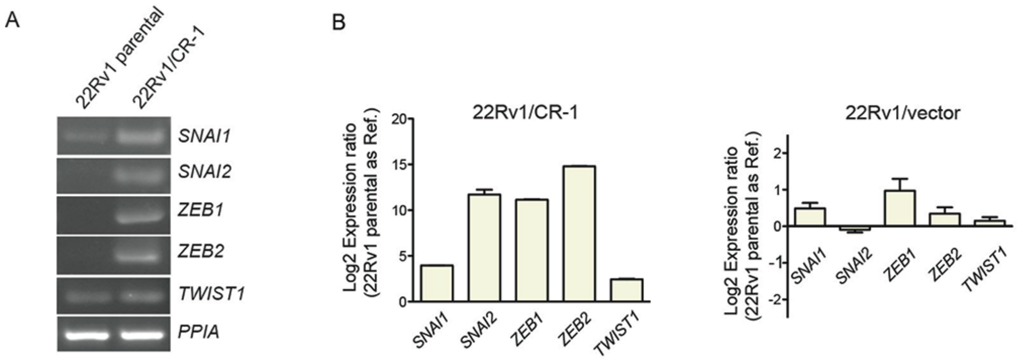

Supplementary Figure S6: Evaluation of *SNAI1, SNAI2, ZEB1*, *ZEB2* and *TWIST1* mRNA levels in 22Rv1 cells and transfected derivatives. **A**, RT-PCR analysis showing increased levels of EMT-inducing TFs *SNAI1, SNAI2, ZEB1*, *ZEB2* and *TWIST1* in 22Rv1/CR-1 cells. **B**, qRT-PCR comparing level expression of these genes in 22Rv1/CR-1 and 22Rv1/vector cells relative to 22Rv1 parental cells. Note that different values were used for the Y axis.

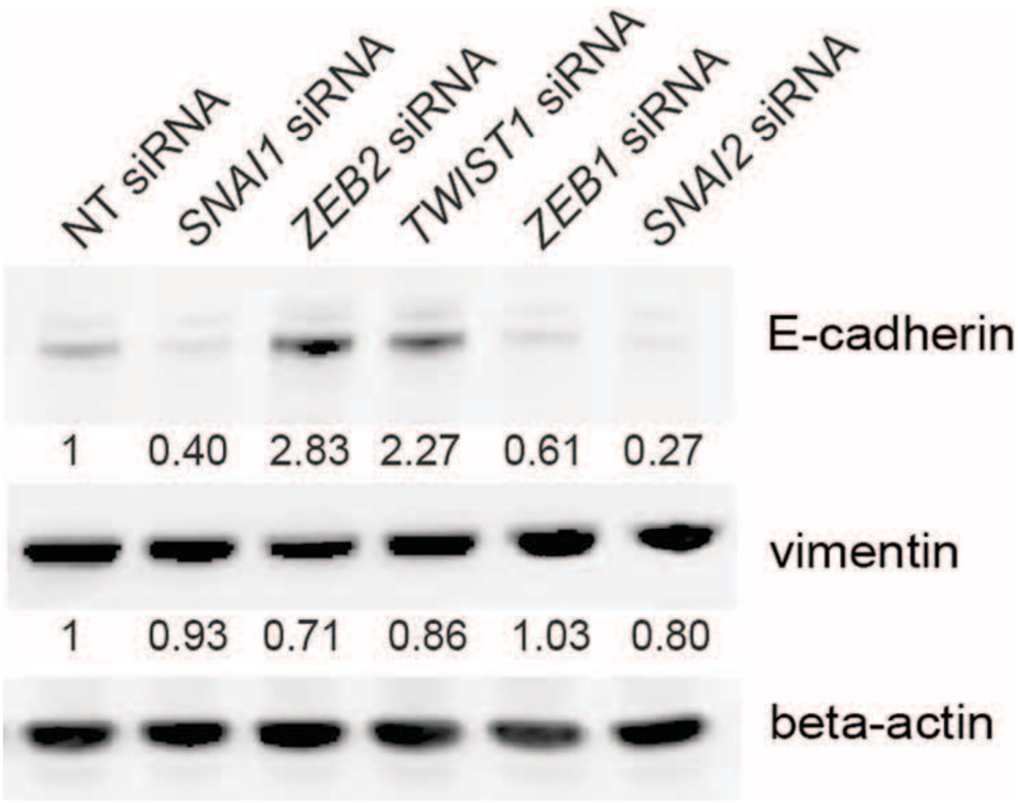

Supplementary Figure S7: knockdown of *TWIST1* and *ZEB2* expression in 22Rv1/CR-1 cells induced changes in expression of E-cadherin and vimentin. 22Rv1/CR-1 cells were treated with non-targeting siRNAs or siRNAs against *ZEB1*, *ZEB2*, *SNAI1*, *SNAI2*, or *TWIST1*, and protein levels of E-cadherin and vimentin were assessed by WB.

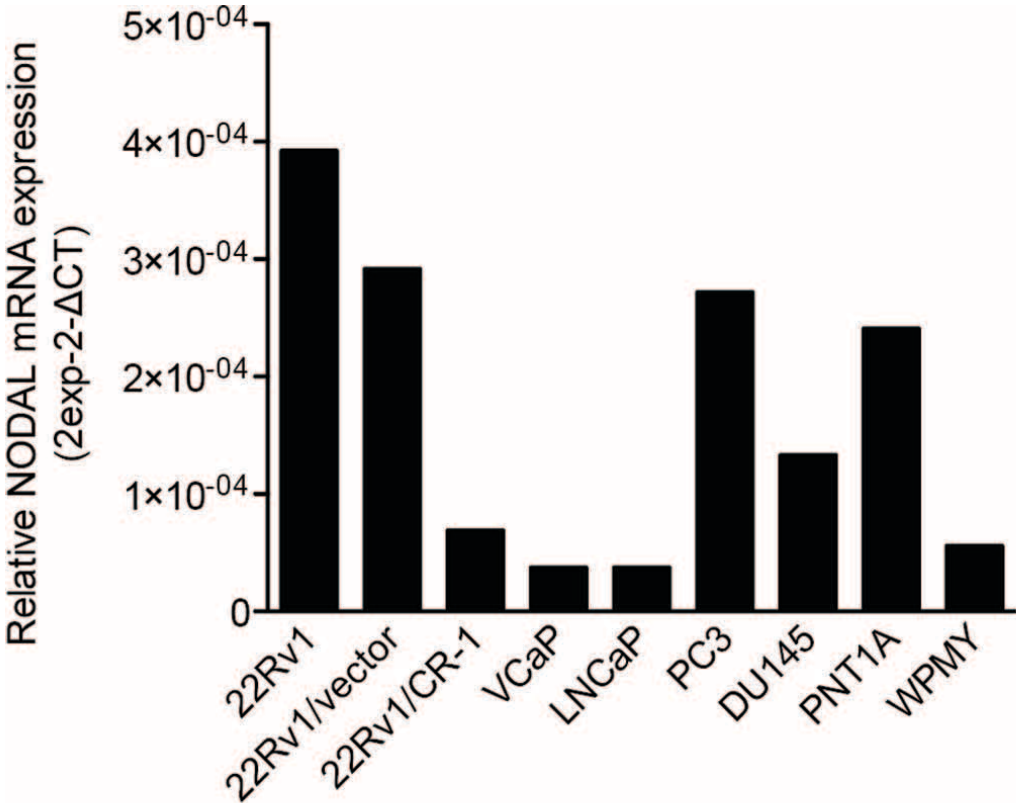

Supplementary Figure S8: *NODAL* transcript expression in a panel of prostate and PCa cell lines. Normalized expression levels are shown (against *RPLP0)*

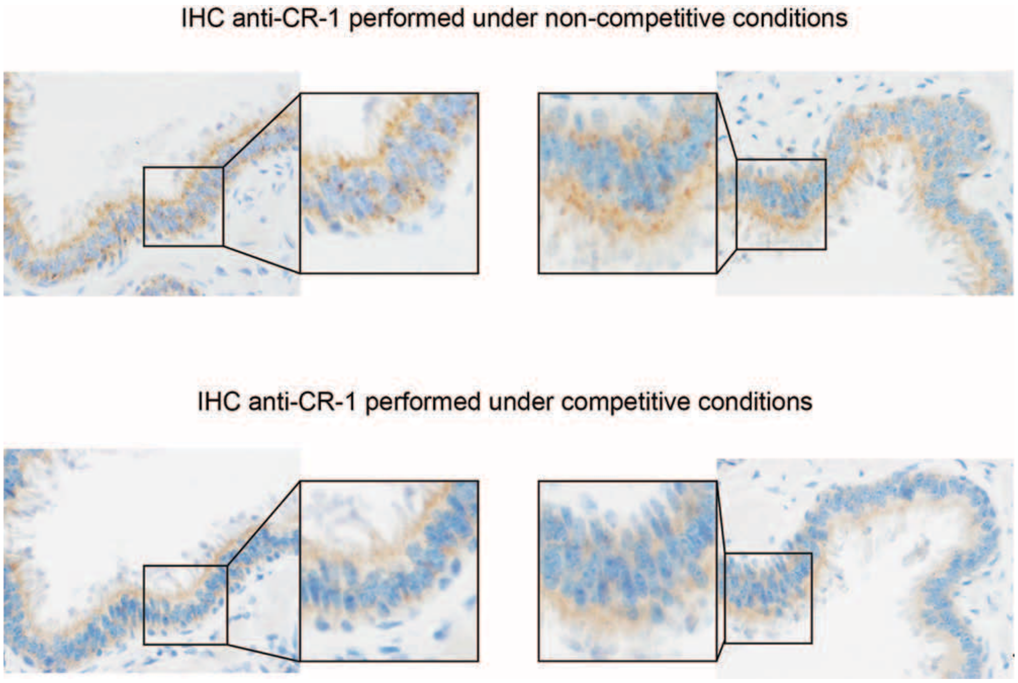

Supplementary Figure S9: IHC on consecutive sections assessing specificity of the anti-CR-1 in human prostate cancer tissues. Immunocompetitive experiment is performed in which anti-human CR-1 antibodies were pre-incubated for 2 ½ hours at 37°C on Elisa plates coated with 1% BSA alone (upper panels, non-competitive condition) or with an additional 300 ng/mL of recombinant human Cripto-1 (R&D Systems)(lower panels). Under non-competitive procedure, IHC reveals positivity with granular staining in neoplastic cells. This type of granular staining was reported earlier and likely corresponds to an enrichment of CR-1 in RE, Golgi and endosomal-like structures [7–9]. Note the absence of staining in the surrounding stroma. Under immunocompetitive conditions (lower panels), a weak staining is observed in the neoplastic cells with a marked diminution of positive granules. Based on this, weak and diffuse staining in the cytoplasm was considered as background, and only specific coarse and granular staining was considered and scored, based on the density of positive granules.

Supplementary Table S1: Information summary from previous studies that investigated CR-1 expression or function in prostate cancer

Reference	*human PCa specimens examined for CR-1*	*Human PCa cell lines or prostate cells examined for CR-1 expression*	*Functional activity of CR-1 and correlations in PCa cell lines*	*Deregulated signaling pathways under CR-1 expression*
Okajima E *et al.* Cancer Lett. 1997 ;111(1–2): 67–70 [1]	9 PCa cases reported as negative for CR-1 (IHC)	nd	nd	nd
Shani G *et al.* Mol Cell Biol. 2008 Jan;28(2): 666–77 [2]	nd	nd	ectopic CR-1 has a potential role in anchorage -dependent and -independent growth when PC3 cells are exposed to TGF-ß1	TGF-beta response/ Smad-2
Cocciadiferro L *et al.* Ann N Y Acad Sci. 2009,1155:257–62 [3]	nd	CR-1 detectable by RT-PCR in LNCaP and PC3 cell lines and by ICC (data not shown)	A seeming increase in CR-1 expression was found in both cell lines under 3D culture conditions at day 4 that decreased at day 6. CR-1 expression correlated with OCT-4 and SUZ12.	nd
Christopher J. Shepherd *et al*. 2008. Prostate, 68: 1007–1024 [4]<br><br>Table S2A available at http://www.crukdmf.icr.ac.uk/array/17/17.html	nd	Expression profiling: CR-1 (*TDGF1*) expression upregulated $CD133^+$ freshly purified benign prostate cells (1.58 fold) compared to the $CD133^-$ benign cells)<br>*TDGF1* was not listed in the published tables depicting deregulated genes in $CD133^+$ cancer cell populations	nd	nd
Lawrence MG *et al.* Prostate. 2011;71(11): 1198–209 [5]	nd	CR-1 found to be expressed at varied levels in LNCaP, 22Rv1, PC3, DU145 and MDA-PCa-2b cell lines. RT-PCR and WB (Rockland Abs) were used	nd	nd

Supplementary Table S2: CR-1 expression and clinico-pathological characteristics of 211 evaluable patients who were treated with radical prostatectomy

	CR-1 (Null to Low)	CR-1 (Intermediate to High)	
No. of patients	131	80	
Age at diagnosis median , years (range)	65 (47.1–75)	65 (50–75)	
Diagnostic PSA median, ng/mL (range):	9 (1.5–99)	9,075 (1.35–53)	
Prostate weight median, grams (range)	50 (20–190)	50 (10–130)	
**Variable**	*n* (%)		
Diagnostic PSA Value			
0–9.9	69 (52.7)	47 (58.75)	Chi-square test ($p = 0.51$)
10–20	45 (34.3)	23 (28.75)	
≥ 20	17 (13.0)	10 (12.5)	
Gleason score			
Gleason score ≤ 6	68 (51.9)	38 (47.5)	Chi-square test ($p = 0.64$)
Gleason 3 + 4	17 (13.0)	9 (11.25)	
Gleason 4 + 3	20 (15.2)	11 (13.75)	
Gleason score ≥ 7	26 (19.9)	22 (27.5)	
Gleason score ≤ 7	105 (80.1)	58 (72.5)	Fisher's exact test
Gleason ≥ 8	26 (19.9)	22 (27.5)	($p = 0.23$)
Tumor stage			
pT2	74 (56.5)	51 (63.75)	Chi-square test ($p = 0.63$)
pT3a	25(19.1)	12 (15)	
pT3b	21 (16.0)	13 (16.25)	
pT4	11 (8.4)	4 (5)	
Biochemical recurrence			
no	104 (80)	50 (62.5)	$p = 0.0064$; OR = 2.4 [1.286–4.481]
yes	26 (20)	30 (37.5)	
Surgical margins			
negative	99 (75.6)	66 (82.5)	Fisher's exact test
postitive	32 (24.4)	14 (17.5)	($p = 0.30$)
Seminal vesicles (VS) status			
SV free	104 (79.4)	65 (81.25)	Fisher's exact test
SV invaded	27 (20.6)	15 (18.75)	($p = 0.85$)
Positive Lymph Node	16 (12.2)	4 (5)	Fisher's exact test ($p = 0.09$)

Supplementary Table S3: Multivariate analysis with a Cox model including Gleason score, pT stage, margin status, Lymph node status and CR-1 expression in prostate cancer cells (n = 136)

Variable	*P* value	HR (95%CI)
Gleason score > 7	0.004	4.93 (1.66–14.64)
pT3-4 cancer	0.822	
Positive margins	0.001	4.55 (1.89–10.96)
Positive lymph nodes	0.001	12.82 (2.83–58.04)
CR-1 expression > 25%	0.006	3.01 (1.37–6.61)

Supplementary Table S4: oligonucleotide primers for RT-PCR

Gene	Accession number	primer	qPCR primer (5′–3′)
RPLP0	NM_001002	Forward	GGCGACCTGGAAGTCCAACT
RPLP0	NM_001002	Reverse	CCATCAGCACCACAGCCTTC
PPIA	NM_021130	Forward	ACCGTGTTCTTCGACATTGC
PPIA	NM_021130	Reverse	GGCATGAATATTGTGGAGGC
TDGF1 (CRIPTO)	NM_003212	Forward	GCAAAGAGAACTGTGGGTCTG
TDGF1 (CRIPTO)	NM_003212	Reverse	GTACGTGCAGACGGTGGTAGT
ZEB1	NM_001128128	Forward	AAGAACTGCTGGGAGGATGACA
ZEB1	NM_001128128	Reverse	CCTCTTCAGGTGCCTCAGGAAAA
ZEB2	NM_014795	Forward	GCAAGAGGCGCAAACAAGC
ZEB2	NM_014795	Reverse	GGGTTGGCAATACCGTCATCC
SNAI1	NM_005985	Forward	CGAAAGGCCTTCAACTGCAAA
SNAI1	NM_005985	Reverse	TGACATCTGAGTGGGTCTGGA
SNAI2	NM_003068	Forward	CTACAGCGAACTGGACACACA
SNAI2	NM_003068	Reverse	TGGAATGGAGCAGCGGTAGT
TWIST1	NM_000474	Forward	CGGAGACCTAGATGTCATTGTTTC
TWIST1	NM_000474	Reverse	CCCACGCCCTGTTTCTTTG
FGFR1	NM_023110.2	Forward	CCTGGTGACAGAGGACAATGT
FGFR1	NM_023110.2	Reverse	GTAGATCCGGTCAAATAATGCCT
FGFR2	NM_000141	Forward	AACGGGAAGGAGTTTAAGCAGG
FGFR2	NM_000141	Reverse	CCCTTGTCAGATGGGACCAC
FGFR3	NM_000142	Forward	AGGCCATCGGCATTGACAAG
FGFR3	NM_000142	Reverse	GCATCGTCTTTCAGCATCTTCAC
FGFR4	NM_002011	Forward	GGGTCCTGCTGAGTGTGC
FGFR4	NM_002011	Reverse	GGGGTAACTGTGCCTATTCG
VIM	NM_003380	Forward	CCTTGAACGCAAAGTGGAATC

Gene	Accession number	primer	qPCR primer (5′–3′)
VIM	NM_003380	Reverse	GACATGCTGTTCCTGAATCTGAG
CDH1	NM_004360	Forward	AGTGCCAACTGGACCATTCA
CDH1	NM_004360	Reverse	TCTTTGACCACCGCTCTCCT
CDH2	NM_001792	Forward	CAACGGGGACTGCACAGATG
CDH2	NM_001792	Reverse	TGTTTGGCCTGGCGTTCTTT
CDH3	NM_001793	Forward	ACGGGGACCATTTTACCATCAC
CDH3	NM_001793	Reverse	CCTCCTGGACCTCAACGACTTT
PAI1 (SERPINE1)	NM_000602	Forward	CACAAATCAGACGGCAGCACT
PAI1 (SERPINE1)	NM_000602	Reverse	CATCGGGCGTGGTGAACTC
CTNNB1	NM_001904	Forward	CCCACTGGCCTCTGATAAAGG
CTNNB1	NM_001904	Reverse	ACGCAAAGGTGCATGATTTG
CD44	NM_000610	Forward	CCATTTTGCCCTTCCATAGCC
CD44	NM_000610	Reverse	CAACCCCCAACCTCAGTGG
FN1	NM_212482	Forward	TGCGAGAGTAAACCTGAAGCTG
FN1	NM_212482	Reverse	ACCCACTCGGTAAGTGTTCCC

REFERENCES

1. Okajima E, Tsutsumi M, Konishi Y. Cripto expression in human urological tumors. Cancer Lett. 1997; 111:67–70
2. Shani G, Fischer WH, Justice NJ, Kelber JA, Vale W, Gray PC. GRP78 and Cripto form a complex at the cell surface and collaborate to inhibit transforming growth factor beta signaling and enhance cell growth. Mol Cell Biol. 2008; 28:666–677.
3. Cocciadiferro L, Miceli V, Kang KS, Polito LM, Trosko JE, Carruba G. Profiling cancer stem cells in androgen-responsive and refractory human prostate tumor cell lines. Ann N Y Acad Sci. 2009; 1155:257–262.
4. Shepherd CJ, Rizzo S, Ledaki I, Davies M, Brewer D, Attard G, de Bono J, Hudson DL. Expression profiling of CD133+ and CD133− epithelial cells from human prostate. Prostate. 2008; 68:1007–1024.
5. Lawrence MG, Margaryan NV, Loessner D, Collins A, Kerr KM, Turner M, Seftor EA, Stephens CR, Lai J, Postovit LM, Clements JA, Hendrix MJ. Reactivation of embryonic Nodal signaling is associated with tumor progression and promotes the growth of prostate cancer cells. Prostate. 2011; 71:1198–1209.
6. Normanno N, De Luca A, Maiello MR, Bianco C, Mancino M, Strizzi L, Arra C, Ciardiello F, Agrawal S, Salomon DS. CRIPTO-1: a novel target for therapeutic intervention in human carcinoma. Int J Oncol. 2004; 25:1013–1020.
7. Blanchet MH, Le Good JA, Oorschot V, Baflast S, Minchiotti G, Klumperman J, Constam DB. Cripto localizes Nodal at the limiting membrane of early endosomes. Sci Signal. 2008; 1:ra13.
8. Blanchet MH, Le Good JA, Mesnard D, Oorschot V, Baflast S, Minchiotti G, Klumperman J, Constam DB. Cripto recruits Furin and PACE4 and controls Nodal trafficking during proteolytic maturation. EMBO J. 2008; 27:2580–2591.
9. Watanabe K, Nagaoka T, Lee JM, Bianco C, Gonzales M, Castro NP, Rangel MC, Sakamoto K, Sun Y, Callahan R, Salomon DS. Enhancement of Notch receptor maturation and signaling sensitivity by Cripto-1. J Cell Biol. 2009; 187:343–353.
